# Disparities in COVID-19 Vaccination Coverage Between Urban and Rural Counties — United States, December 14, 2020–January 31, 2022

**DOI:** 10.15585/mmwr.mm7109a2

**Published:** 2022-03-04

**Authors:** Ryan Saelee, Elizabeth Zell, Bhavini Patel Murthy, Patricia Castro-Roman, Hannah Fast, Lu Meng, Lauren Shaw, Lynn Gibbs-Scharf, Terence Chorba, LaTreace Q. Harris, Neil Murthy

**Affiliations:** ^1^Immunization Services Division, National Center for Immunization and Respiratory Diseases, CDC; ^2^CDC COVID-19 Emergency Response Team; ^3^Stat-Epi Associates, Inc., Ponte Vedra Beach, Florida; ^4^Division of Global Migration and Quarantine, National Center for Emerging and Zoonotic Infectious Diseases, CDC; ^5^General Dynamics Information Technology, Inc., Falls Church, Virginia; ^6^Division of Tuberculosis Elimination, National Center for HIV, Viral Hepatitis, STD, and TB Prevention, CDC.

Higher COVID-19 incidence and mortality rates in rural than in urban areas are well documented (*1*). These disparities persisted during the B.1.617.2 (Delta) and B.1.1.529 (Omicron) variant surges during late 2021 and early 2022 (*1*,*2*). Rural populations tend to be older (aged ≥65 years) and uninsured and are more likely to have underlying medical conditions and live farther from facilities that provide tertiary medical care, placing them at higher risk for adverse COVID-19 outcomes (*2*). To better understand COVID-19 vaccination disparities between urban and rural populations, CDC analyzed county-level vaccine administration data among persons aged ≥5 years who received their first dose of either the BNT162b2 (Pfizer-BioNTech) or mRNA-1273 (Moderna) COVID-19 vaccine or a single dose of the Ad.26.COV2.S (Janssen [Johnson & Johnson]) COVID-19 vaccine during December 14, 2020–January 31, 2022, in 50 states and the District of Columbia (DC). COVID-19 vaccination coverage with ≥1 doses in rural areas (58.5%) was lower than that in urban counties (75.4%) overall, with similar patterns across age groups and sex. Coverage with ≥1 doses varied among states: 46 states had higher coverage in urban than in rural counties, one had higher coverage in rural than in urban counties. Three states and DC had no rural counties; thus, urban-rural differences could not be assessed. COVID-19 vaccine primary series completion was higher in urban than in rural counties. However, receipt of booster or additional doses among primary series recipients was similarly low between urban and rural counties. Compared with estimates from a previous study of vaccine coverage among adults aged ≥18 years during December 14, 2020–April 10, 2021, these urban-rural disparities among those now eligible for vaccination (aged ≥5 years) have increased more than twofold through January 2022, despite increased availability and access to COVID-19 vaccines. Addressing barriers to vaccination in rural areas is critical to achieving vaccine equity, reducing disparities, and decreasing COVID-19–related illness and death in the United States (*2*).

Data on COVID-19 vaccine doses administered in the United States are reported to CDC by jurisdictions, pharmacies, and federal entities through immunization information systems (IISs),* the Vaccine Administration Management System (VAMS),^†^ or through direct data submission.^§^ Persons aged ≥5 years with a valid county of residence in one of the 50 states or DC who received their first dose of a COVID-19 vaccine^¶^ during December 14, 2020–January 31, 2022, and whose deidentified data were reported to CDC were included in the analysis.** Urban-rural comparisons could not be made for three states (Delaware, New Jersey, and Rhode Island) and DC because they only had urban counties. In addition, eight counties in California with population size <20,000 were excluded because they have data-sharing restrictions on county-level information reported to CDC. Vaccine doses administered to persons residing in U.S. territories and freely associated states were also excluded because jurisdictional divisions could not be mapped to urban-rural classifications at the county level.

Receipt of the first dose of COVID-19 vaccine was matched by county of residence to one of six urban-rural categories according to the 2013 National Center for Health Statistics Urban-Rural Classification Scheme (*3*). To further classify counties into two categories (urban versus rural), four of these six categories (large central metropolitan, large fringe metropolitan, medium metropolitan, and small metropolitan) were combined into an urban category, and two (micropolitan and noncore) were combined into a rural category (*3*).

Vaccination coverage for persons aged ≥5 years who received ≥1 doses of a 2-dose COVID-19 primary vaccination series or a single dose of the Janssen COVID-19 vaccine was calculated overall and by age group (5–11, 12–17, 18–64, and ≥65 years), sex, jurisdiction, and urban-rural classification (two- and six-level). Population size was obtained by county, age group, and sex from the U.S. Census Bureau’s 2020 Population Estimates Program (*4*). Because only the first dose of a 2-dose primary vaccination series or the single dose for Janssen vaccine was analyzed, the total number of doses per county was capped at the county's population size.^††^ Primary series completion^§§^ was also calculated and stratified by urban-rural classification. Among those aged ≥12 years who had completed their primary COVID-19 vaccination series, the proportions eligible for a booster dose and with sufficient time to receive it,^¶¶^ as well as the proportions of eligible persons who did and did not receive a booster dose, were calculated and stratified by urban-rural classification. Tests for statistical significance were not conducted because the data represent the U.S. population (excluding eight counties in California) and were not based on population samples. All analyses were conducted using SAS software (version 9.4; SAS Institute). This activity was reviewed by CDC and was conducted consistent with applicable federal law and CDC policy.***

Overall, during December 14, 2020–January 31, 2022, rural counties had lower first-dose vaccination coverage (58.5%) than did urban counties (75.4%) (Table 1). Females and males had lower first-dose coverage in rural counties (61.4% and 55.7%, respectively) than in urban counties (77.6% and 73.2%, respectively). Among all age groups, vaccination coverage with ≥1 doses was lower in rural counties, with the largest absolute difference (26.2 percentage points) among those aged 12–17 years (38.7% rural, 64.9% urban) and the largest relative difference among those aged 5–11 years (14.7% rural, 30.5% urban). Across jurisdictions, vaccination coverage with ≥1 doses varied by urban-rural classification. Among jurisdictions for which the urban-rural classification could be calculated, 46 jurisdictions had higher coverage in urban than rural counties, and one jurisdiction (Arizona) had higher coverage in rural than urban counties (Table 2). Primary series completion was lower in rural counties (52.1%) than in urban counties (66.2%) (Table 3). Receipt of booster or additional doses among those eligible was similar between urban (50.4%) and rural counties (49.7%).

**TABLE 1:** COVID-19 vaccination coverage for persons aged ≥5 years who have received their first dose of the Moderna or Pfizer-BioNTech vaccine or a single dose of the Janssen (Johnson & Johnson) vaccine,* by sex, age group, and urban-rural classification^†^ — United States, December 14, 2020–January 31, 2022

Characteristic	No. (%)								
	Overall	Six-level urban-rural classification						Two-level urban-rural classification	
		Large central metropolitan	Large fringe metropolitan	Medium metropolitan	Small metropolitan	Micropolitan	Noncore	Urban	Rural
**Total**	**226,621,879 (73.1)**	76,387,928 (80.4)	59,624,160 (76.1)	47,054,083 (72.2)	18,185,028 (64.4)	15,549,920 (60.4)	9,820,760 (55.8)	201,251,199 (75.4)	25,370,680 (58.5)
**Sex**									
Male	**107,681,923 (70.7)**	36,514,502 (78.7)	28,228,944 (73.6)	22,236,671 (69.6)	8,633,027 (61.9)	7,393,069 (57.6)	4,675,710 (52.8)	95,613,144 (73.2)	12,068,779 (55.7)
Female	**118,939,956 (75.4)**	39,873,426 (82.0)	31,395,216 (78.6)	24,817,412 (74.7)	9,552,001 (66.8)	8,156,851 (63.2)	5,145,050 (58.8)	105,638,055 (77.6)	13,301,901 (61.4)
**Age group, yrs**									
5–11	**8,046,457 (28.4)**	3,007,534 (34.7)	2,363,850 (32.5)	1,591,017 (26.3)	517,209 (20.4)	373,368 (16.1)	193,479 (12.5)	7,479,610 (30.5)	566,847 (14.7)
12–17	**15,398,653 (61.3)**	5,419,083 (73.0)	4,346,283 (65.4)	3,203,152 (59.9)	1,080,652 (48.5)	866,250 (41.7)	483,233 (34.2)	14,049,170 (64.9)	1,349,483 (38.7)
18–64	**152,499,838 (75.9)**	54,096,094 (84.4)	40,059,543 (79.1)	30,972,242 (74.4)	11,706,128 (65.3)	9,782,658 (61.0)	5,883,173 (55.7)	136,834,007 (78.5)	15,665,831 (58.9)
≥65	**50,676,931 (91.1)**	13,865,217 (93.5)	12,854,484 (93.7)	11,287,672 (93.0)	4,881,039 (87.6)	4,527,644 (85.4)	3,260,875 (80.2)	42,888,412 (92.7)	7,788,519 (83.2)

* Excludes doses with state of residence reported as a territory, freely associated state, or county of residence in California with population <20,000. Completeness of county data varied by jurisdiction.

^†^ First doses of COVID-19 vaccine were matched by county of residence to one of six urban-rural categories according to the 2013 National Center for Health Statistics Urban-Rural Classification Scheme (https://www.cdc.gov/nchs/data/series/sr_02/sr02_166.pdf). To further classify counties into two categories (urban versus rural), four of these six categories were combined into urban areas (large central metropolitan, large fringe metropolitan, medium metropolitan, and small metropolitan), and two were combined into rural areas (micropolitan and noncore).

**TABLE 2:** COVID-19 vaccination coverage for persons aged ≥5 years who have received their first dose of the Moderna or Pfizer-BioNTech vaccine, or a single dose of the Janssen (Johnson & Johnson) vaccine, by jurisdiction* and urban-rural classification^†^ — December 14, 2020–January 31, 2022

Jurisdiction	Vaccination coverage, no. (%)									
	Overall no. (%) with available county-level data	Six-level urban-rural classification							Two-level urban-rural classification	
		Large central metropolitan	Large fringe metropolitan	Medium metropolitan	Small metropolitan	Micropolitan	Noncore	Urban		Rural
**All**	**226,621,879 (73.1)**	**76,387,928 (80.4)**	**59,624,160 (76.1)**	**47,054,083 (72.2)**	**18,185,028 (64.4)**	**15,549,920 (60.4)**	**9,820,760 (55.8)**	**201,251,199 (75.4)**		**25,370,680 (58.5)**
Alabama	2,757,503 (59.6)	455,302 (74.2)	221,709 (47.0)	823,384 (66.2)	696,028 (56.4)	267,428 (53.9)	293,652 (51.5)	2,196,423 (61.6)		561,080 (52.6)
Alaska	457,999 (68.1)	—^§^	—^§^	254,919 (68.8)	63,589 (71.6)	35,442 (82.1)	104,049 (61.1)	318,508 (69.4)		139,491 (65.4)
Arizona	4,902,381 (70.1)	2,893,327 (67.2)	278,396 (61.2)	815,028 (81.1)	628,677 (70.1)	203,258 (85.9)	83,695 (86.7)	4,615,428 (69.3)		286,953 (86.1)
Arkansas	1,648,929 (58.0)	—^§^	24,271 (55.1)	887,427 (63.8)	175,592 (49.6)	282,910 (53.4)	278,729 (53.1)	1,087,290 (60.7)		561,639 (53.3)
California	30,179,535 (81.6)	19,997,305 (85.1)	3,877,313 (78.7)	5,036,170 (75.1)	790,163 (70.7)	363,889 (68.2)	114,695 (67.3)	29,700,951 (81.9)		478,584 (68.0)
Colorado	4,185,782 (76.3)	586,633 (84.6)	1,679,502 (78.9)	1,274,961 (76.2)	183,689 (59.7)	276,592 (72.7)	184,405 (61.6)	3,724,785 (77.5)		460,997 (67.8)
Connecticut	3,088,922 (91.5)	748,852 (88.9)	259,254 (86.5)	1,927,679 (93.4)	—^§^	153,137 (88.9)	—^§^	2,935,785 (91.6)		153,137 (88.9)
Delaware	742,926 (79.7)	—^§^	448,379 (84.6)	180,672 (78.6)	113,875 (66.0)	—^§^	—^§^	742,926 (79.7)		—^§^
District of Columbia	640,686 (95.8)	640,686 (95.8)	—^§^	—^§^	—^§^	—^§^	—^§^	640,686 (95.8)		—^§^
Florida	15,798,256 (76.7)	5,897,602 (82.8)	4,508,848 (78.2)	4,160,855 (71.2)	854,221 (72.5)	215,083 (61.3)	161,647 (48.4)	15,421,526 (77.4)		376,730 (55.0)
Georgia	5,818,300 (57.8)	676,861 (66.5)	2,805,562 (59.7)	685,239 (60.1)	818,217 (54.8)	470,916 (49.5)	361,505 (47.6)	4,985,879 (59.7)		832,421 (48.7)
Hawaii	1,166,364 (88.2)	—^§^	—^§^	833,497 (92.1)	127,790 (80.7)	205,077 (78.9)	—^§^	961,287 (90.4)		205,077 (78.9)
Idaho	989,974 (57.8)	—^§^	—^§^	459,601 (63.4)	237,160 (54.1)	227,660 (55.3)	65,553 (47.8)	696,761 (59.9)		293,213 (53.4)
Illinois	9,268,865 (78.2)	4,100,438 (85.3)	3,117,807 (80.6)	582,524 (68.8)	666,813 (67.6)	491,120 (61.4)	310,163 (56.3)	8,467,582 (80.6)		801,283 (59.3)
Indiana	3,927,951 (62.0)	591,225 (65.9)	1,405,574 (68.3)	582,136 (63.0)	647,621 (59.3)	490,631 (52.2)	210,764 (49.1)	3,226,556 (64.9)		701,395 (51.3)
Iowa	1,995,392 (67.2)	—^§^	—^§^	847,442 (73.0)	438,094 (69.6)	286,111 (62.4)	423,745 (58.6)	1,285,536 (71.8)		709,856 (60.1)
Kansas	1,889,944 (69.2)	—^§^	708,348 (84.2)	399,033 (65.6)	285,569 (65.9)	300,857 (60.9)	196,137 (55.5)	1,392,950 (74.0)		496,994 (58.6)
Kentucky	2,679,957 (63.7)	558,304 (77.5)	429,089 (65.4)	474,368 (69.0)	247,934 (58.1)	455,933 (56.4)	514,329 (56.6)	1,709,695 (68.7)		970,262 (56.5)
Louisiana	2,641,765 (60.7)	303,080 (82.5)	590,214 (71.3)	958,914 (58.3)	437,889 (53.3)	191,525 (52.8)	160,143 (49.1)	2,290,097 (62.6)		351,668 (51.0)
Maine	1,108,287 (86.1)	—^§^	—^§^	489,231 (94.5)	198,983 (80.5)	94,230 (80.6)	325,843 (80.5)	688,214 (90.0)		420,073 (80.5)
Maryland	4,826,509 (84.7)	418,821 (76.1)	3,967,335 (87.9)	215,045 (68.3)	124,839 (71.5)	49,943 (76.4)	50,526 (65.3)	4,726,040 (85.1)		100,469 (70.4)
Massachusetts	5,706,211 (87.2)	697,974 (91.7)	3,659,320 (91.9)	1,174,555 (85.1)	113,507 (34.9)	60,355 (71.7)	500 (4.7)	5,645,356 (87.6)		60,855 (64.2)
Michigan	5,826,988 (61.9)	1,381,910 (61.6)	1,823,505 (64.6)	990,899 (63.7)	640,707 (59.3)	626,474 (58.3)	363,493 (57.6)	4,837,021 (62.8)		989,967 (58.1)
Minnesota	3,830,405 (72.1)	1,387,321 (81.5)	1,114,942 (69.0)	164,688 (73.9)	425,982 (70.7)	416,618 (66.3)	320,854 (59.2)	3,092,933 (74.7)		737,472 (63.0)
Mississippi	1,679,842 (60.3)	—^§^	162,000 (63.8)	583,623 (63.8)	82,810 (58.6)	510,014 (58.7)	341,395 (56.3)	828,433 (63.2)		851,409 (57.7)
Missouri	3,582,610 (61.9)	688,006 (73.1)	1,557,395 (69.1)	256,709 (54.9)	386,917 (57.1)	333,686 (49.7)	359,897 (46.6)	2,889,027 (66.6)		693,583 (48.1)
Montana	632,670 (62.0)	—^§^	—^§^	—^§^	228,239 (64.0)	202,588 (62.9)	201,843 (59.1)	228,239 (64.0)		404,431 (60.9)
Nebraska	1,117,283 (61.8)	—^§^	—^§^	797,578 (73.1)	53,722 (52.7)	129,210 (42.5)	136,773 (43.9)	851,300 (71.3)		265,983 (43.2)
Nevada	2,098,894 (71.1)	1,566,670 (72.0)	—^§^	345,349 (76.1)	40,393 (76.4)	130,281 (54.4)	16,201 (53.3)	1,952,412 (72.7)		146,482 (54.3)
New Hampshire	1,193,635 (91.6)	—^§^	393,877 (93.2)	361,471 (91.0)	—^§^	394,069 (90.4)	44,218 (93.2)	755,348 (92.1)		438,287 (90.7)
New Jersey	7,314,550 (87.4)	1,750,970 (92.5)	4,786,993 (86.2)	603,833 (86.7)	172,754 (76.7)	—^§^	—^§^	7,314,550 (87.4)		—^§^
New Mexico	1,604,088 (80.7)	—^§^	—^§^	723,609 (82.7)	413,490 (88.1)	406,316 (72.9)	60,673 (70.6)	1,137,099 (84.6)		466,989 (72.6)
New York	15,684,228 (86.0)	8,401,387 (90.2)	—^§^	1,353,652 (78.8)	627,694 (79.5)	634,961 (69.1)	237,481 (65.1)	14,811,786 (87.4)		872,442 (68.0)
North Carolina	7,803,797 (78.1)	1,935,906 (91.3)	956,059 (70.6)	2,815,151 (79.9)	657,630 (73.8)	1,036,172 (68.7)	402,879 (67.6)	6,364,746 (80.7)		1,439,051 (68.4)
North Dakota	429,616 (60.3)	—^§^	—^§^	—^§^	236,356 (65.4)	88,397 (52.1)	104,863 (57.9)	236,356 (65.4)		193,260 (55.1)
Ohio	6,925,142 (62.9)	2,244,625 (71.1)	1,540,587 (65.1)	1,790,442 (63.5)	241,601 (52.5)	920,736 (51.6)	187,151 (44.7)	5,817,255 (66.1)		1,107,887 (50.3)
Oklahoma	2,502,179 (67.1)	618,215 (82.7)	381,909 (65.1)	695,416 (67.7)	89,297 (75.5)	440,229 (59.1)	277,113 (55.1)	1,784,837 (72.0)		717,342 (57.5)
Oregon	3,093,299 (76.9)	708,636 (91.4)	903,139 (80.5)	570,920 (73.6)	482,396 (69.0)	361,879 (65.8)	66,329 (67.6)	2,665,091 (79.0)		428,208 (66.1)
Pennsylvania	9,420,480 (77.9)	2,315,543 (88.2)	3,001,055 (85.3)	2,588,460 (74.7)	724,186 (64.7)	583,977 (59.2)	207,259 (54.6)	8,629,244 (80.4)		791,236 (58.0)
Rhode Island	850,640 (84.8)	494,329 (82.3)	356,311 (88.5)	—^§^	—^§^	—^§^	—^§^	850,640 (84.8)		—^§^
South Carolina	3,115,559 (63.2)	—^§^	246,655 (61.9)	2,108,729 (63.5)	352,130 (69.4)	240,259 (56.9)	167,786 (60.3)	2,707,514 (64.1)		408,045 (58.3)
South Dakota	602,302 (72.4)	—^§^	—^§^	—^§^	315,121 (76.2)	152,801 (69.7)	134,380 (67.2)	315,121 (76.2)		287,181 (68.6)
Tennessee	3,996,653 (61.7)	1,100,667 (72.4)	804,507 (61.3)	1,057,733 (64.2)	307,777 (56.0)	422,790 (50.9)	303,179 (49.1)	3,270,684 (65.0)		725,969 (50.1)
Texas	17,422,544 (63.6)	8,869,150 (68.5)	3,451,074 (62.3)	2,947,901 (68.8)	827,824 (47.8)	765,416 (49.9)	561,179 (41.4)	16,095,949 (65.7)		1,326,595 (45.9)
Utah	2,175,366 (72.3)	876,508 (80.8)	45,977 (66.7)	870,732 (70.0)	184,278 (62.8)	116,968 (65.5)	80,903 (58.2)	1,977,495 (73.5)		197,871 (62.3)
Vermont	510,091 (85.7)	—^§^	—^§^	—^§^	185,762 (88.2)	194,964 (84.5)	129,365 (84.2)	185,762 (88.2)		324,329 (84.4)
Virginia	5,802,571 (71.8)	890,652 (71.6)	3,414,756 (76.5)	393,085 (61.8)	513,580 (67.8)	142,629 (58.1)	447,869 (60.8)	5,212,073 (73.4)		590,498 (60.1)
Washington	5,661,229 (78.2)	1,936,717 (90.1)	1,596,654 (75.5)	971,606 (70.8)	652,690 (74.4)	391,498 (68.1)	112,064 (71.4)	5,157,667 (79.2)		503,562 (68.8)
West Virginia	1,098,194 (64.8)	—^§^	41,649 (76.3)	206,145 (66.3)	462,288 (67.5)	173,245 (62.5)	214,867 (58.7)	710,082 (67.6)		388,112 (60.4)
Wisconsin	3,909,379 (71.0)	654,306 (74.2)	635,142 (71.5)	793,672 (80.9)	927,701 (69.6)	469,365 (63.6)	429,193 (62.6)	3,010,821 (73.8)		898,558 (63.1)
Wyoming	315,207 (57.5)	—^§^	—^§^	—^§^	101,453 (59.6)	142,281 (62.4)	71,473 (47.6)	101,453 (59.6)		213,754 (56.5)

* Excludes doses with state of residence reported as a territory, freely associated state, or county of residence in California with population <20,000. Completeness of county data varied by jurisdiction.

^†^ First doses of COVID-19 vaccine were matched by county of residence to one of six urban-rural categories according to the 2013 National Center for Health Statistics Urban-Rural Classification Scheme (https://www.cdc.gov/nchs/data/series/sr_02/sr02_166.pdf). To further classify counties into two categories (urban versus rural), four of these six categories were combined into urban areas (large central metropolitan, large fringe metropolitan, medium metropolitan, and small metropolitan), and two were combined into rural areas (micropolitan and noncore).

^§^ State has no counties at this level of urban-rural classification.

**TABLE 3:** COVID-19 vaccine series completion* and receipt of booster or additional dose among eligible population,^†^ by urban-rural classification^§^ — United States, December 14, 2020–January 31, 2022

Characteristic	No. (%)								
	Overall	Six-level urban-rural classification						Two-level urban-rural classification	
		Large central metropolitan	Large fringe metropolitan	Medium metropolitan	Small metropolitan	Micropolitan	Noncore	Urban	Rural
Completed series*	199,221,855 (64.2)	66,993,451 (70.5)	52,606,393 (67.2)	40,925,558 (62.8)	16,107,552 (57.0)	13,783,465 (53.6)	8,805,436 (50.0)	176,632,954 (66.2)	22,588,901 (52.1)
**Eligible for booster dose^†^**	151,089,493	49,090,381	39,909,203	32,033,567	12,472,784	10,892,636	6,690,922	133,505,935	17,583,558
**Received booster or additional dose**									
Yes	75,983,349 (50.3)	24,689,986 (50.3)	20,439,141 (51.2)	15,878,242 (49.6)	6,244,751 (50.1)	5,343,170 (49.1)	3,388,059 (50.6)	67,252,120 (50.4)	8,731,229 (49.7)
No	75,106,144 (49.7)	24,400,395 (49.7)	19,470,062 (48.8)	16,155,325 (50.4)	6,228,033 (49.9)	5,549,466 (50.9)	3,302,863 (49.4)	66,253,815 (49.6)	8,852,329 (50.3)

* Persons aged ≥5 years who received a single dose of Janssen (Johnson & Johnson) vaccine or 2 doses of an mRNA vaccine (Pfizer-BioNTech or Moderna). This includes those who received the same vaccine type for both mRNA vaccine doses, as well as those who received heterologous products for the first and second dose (e.g., Pfizer-BioNTech for first dose and Moderna for the second dose, or vice versa). Excludes doses with state of residence reported as a territory, freely associated state, or county of residence in California with population <20,000. Completeness of county data varied by jurisdiction.

^†^ Eligible population is defined as persons aged ≥12 years who completed a primary COVID-19 vaccination series and were eligible to receive a booster or additional primary dose by the end of the analysis period, January 31, 2022. For Pfizer-BioNTech and Moderna, the primary series must have been completed by August 31, 2021 (i.e., ≥5 months earlier); for Janssen recipients, 1 dose must have been received by December 1, 2021 (i.e., ≥2 months earlier). Excludes residents in Texas and persons aged <18 years from Idaho.

^§^ Doses of COVID-19 vaccine were matched by county of residence to one of six urban-rural categories according to the 2013 National Center for Health Statistics Urban-Rural Classification Scheme (https://www.cdc.gov/nchs/data/series/sr_02/sr02_166.pdf). To further classify counties into two categories (urban versus rural), four of these six categories were combined into urban areas (large central metropolitan, large fringe metropolitan, medium metropolitan, and small metropolitan), and two were combined into rural areas (micropolitan and noncore).

## Discussion

Across the United States, COVID-19 vaccination coverage was lower in rural counties than in urban counties, and this disparity persisted across sex and age groups. Compared with estimates from a previous study of vaccine coverage among adults aged ≥18 years during December 14, 2020–April 10, 2021, these urban-rural disparities among those now eligible (persons aged ≥5 years) for vaccination have increased overall and across sex and age groups, despite increased availability and access to COVID-19 vaccines (*5*). During December 14, 2020–April 10, 2021, urban-rural differences in first-dose COVID-19 vaccination coverage among adults aged ≥18 years were 6.8 percentage points (*5*); this gap has increased more than twofold to 16.9 percentage points in the current analysis among persons aged ≥5 years.

Various factors might have contributed to these increasing disparities. First, access to health care remains challenging in rural counties. Before the pandemic, persons in rural areas were more likely to report not having enough health care providers or hospitals to serve the community compared with persons living in urban areas, which might pose access issues for rural Americans seeking COVID-19 vaccination (*6*). Second, variations in views regarding the seriousness of COVID-19 infection and intention to implement COVID-19 prevention strategies exist and are often shaped by sociocultural identities and political ideologies that vary across the urban-rural continuum (*7*). Third, vaccine hesitancy has been historically higher in rural^†††^ than urban areas for routinely recommended vaccines and continues to drive lower COVID-19 vaccination coverage in rural areas. Adults in rural areas were nearly three times as likely to report that they “definitely won’t” get a COVID-19 vaccine than were those in urban areas (*8*). Targeted efforts are critical to increase vaccine confidence to address gaps in vaccination coverage between urban and rural communities.

Similar factors have also affected pediatric COVID-19 vaccination coverage. Parents in rural communities were approximately twice as likely to state that their child will “definitely not” get a COVID-19 vaccine compared with those in urban communities (*8*). Notably, 76% of parents in rural areas indicated that their trusted source of vaccination information for their children is their health care provider. However, nearly 40% of rural parents reported that their child’s pediatrician did not recommend a COVID-19 vaccine, compared with only 8% of parents in urban communities (*8*). Health care providers remain a trusted source of information for parents, and vaccine recommendations from a health care provider are strong predictors of COVID-19 vaccination (*9*). This reported disparity between urban and rural pediatricians highlights the importance of partnering with health care providers and provider organizations to reduce vaccine hesitancy and increase vaccination coverage. Ongoing joint efforts by the CDC, local and state health departments, and other local partners through the Vaccinate with Confidence^§§§^ initiative are designed to enhance trust and vaccine confidence in rural areas.

Some exceptions to the general trends were observed in this study. Although vaccination coverage was nearly universally higher in urban than rural counties, Arizona was the only state where coverage in rural counties was higher than that in urban counties; the reasons for this finding are not well understood. Rapid research will be important to identify and implement innovative approaches to bridge the gap in coverage between urban and rural counties. Despite pronounced urban-rural differences being noted in first dose COVID-19 vaccination coverage, receipt of booster or additional doses among those eligible was similarly low in urban and rural counties. Although booster doses for adolescents aged 12–17 years were authorized for only approximately 3 weeks during this study period (which might account for low booster dose coverage in this age group), all other age groups had more time to receive booster vaccination. The low booster dose coverage in urban and rural counties highlights the importance of developing and implementing innovative strategies to promote COVID-19 vaccines among all persons who are eligible for booster or additional doses and to receive these doses at the recommended intervals.

The findings in this report are subject to at least four limitations. First, eight counties with population size <20,000 in California were excluded, which might minimally bias coverage estimates. Second, race and ethnicity were unknown for approximately 35% of persons; therefore, vaccination coverage could not be estimated based on race and ethnicity. Third, booster doses could not be distinguished from additional primary doses because of absence of information on the immunocompromise status of vaccine recipients, which can thereby affect the interpretation of these findings. Finally, the National Center for Health Statistics Urban-Rural Classification was developed in 2013, and counties once classified as rural in 2013 might no longer be rural in 2022.

Addressing barriers to vaccination in rural areas is critical to achieving vaccine equity, reducing disparities, and decreasing COVID-19–related illness and death in the United States. Public health practitioners could focus on collaborating with health care providers, pharmacies, schools, community-based organizations, faith leaders, and local employers^¶¶¶^ to improve vaccine confidence, ensure equitable vaccine access, and encourage staying up to date with recommended COVID-19 vaccinations in rural communities (*10*).

**Summary**

**What is already known about this topic?**

COVID-19 incidence and mortality are higher in rural than in urban communities. Disparities in COVID-19 vaccination coverage between urban and rural communities have been recognized.

**What is added by this report?**

COVID-19 vaccination coverage with the first dose of the primary vaccination series was lower in rural (58.5%) than in urban counties (75.4%); disparities have increased more than twofold since April 2021. Receipt of booster or additional doses was similarly low in both rural and urban counties.

**What are the implications for public health practice?**

Addressing barriers to vaccination in rural areas is critical to achieving vaccine equity, reducing disparities, and decreasing COVID-19–related illness and death in the United States.

## Footnotes

* IISs are confidential, computerized, population-based systems that collect and consolidate vaccination data from providers in 64 public health jurisdictions nationwide and can be used to track administered vaccines and measure vaccination coverage. The 64 IIS jurisdictions comprise the 50 U.S. states, five U.S. territories (American Samoa, Guam, Northern Mariana Islands, Puerto Rico, and U.S. Virgin Islands), three freely associated states (Federated States of Micronesia, Marshall Islands, and Palau), and six local jurisdictions (Chicago, Illinois; Houston, Texas; New York, New York; Philadelphia, Pennsylvania; San Antonio, Texas; and Washington, DC).

†
https://www.cdc.gov/vaccines/covid-19/reporting/vams/program-information.html

§
https://www.cdc.gov/vaccines/covid-19/reporting/overview/IT-systems.html

¶ Includes the first dose of a 2-dose vaccination series (Pfizer-BioNTech or Moderna) as well as a single dose of the Janssen vaccine.

** Providers are required to document vaccination in their medical records within 24 hours of administration and in their jurisdiction’s IISs within 72 hours of administration. A total of 5 days of observation were included to account for any delays in reporting and transmission of records to CDC.

†† For statistical analysis, the number of doses per county was capped at the county’s population size minus one for a maximum vaccination coverage of 100%.

§§ Primary series completion is defined as receiving either both doses of a 2-dose mRNA COVID-19 vaccination series (Pfizer-BioNTech or Moderna) or a single dose of the Janssen vaccine. Series completion includes receipt of the same vaccine type for both mRNA doses or mismatched products for the first and second dose (e.g., Pfizer-BioNTech for the first dose and Moderna for the second dose, or vice versa).

¶¶ Eligible population is defined as persons aged ≥12 years who completed a primary COVID-19 vaccination series and were eligible to receive a booster or additional primary dose by the end of the analysis period, January 31, 2022. Those aged 12–17 years were eligible to receive their booster dose beginning January 5, 2022, and persons aged ≥18 years were eligible beginning November 19, 2021. These differences in eligibility dates were accounted for in analyses by restricting data to these eligibility dates by age. For Pfizer-BioNTech and Moderna vaccines, the primary series must have been completed by August 31, 2021 (i.e., ≥5 months earlier); for Janssen recipients, 1 dose must have been received by December 1, 2021 (i.e., ≥2 months earlier).

*** 45 C.F.R. part 46.102(l)(2); 21 C.F.R. part 56; 42 U.S.C. Sect. 241(d); 5 U.S.C. Sect. 552a; 44 U.S.C. Sect. 3501 et seq.

†††
https://www.ruralhealthinfo.org/rural-monitor/increasing-vaccination-rates/

§§§
https://www.cdc.gov/vaccines/covid-19/vaccinate-with-confidence/strategy.html

¶¶¶
https://www.cdc.gov/coronavirus/2019-ncov/vaccines/recommendations/essentialworker/workplace-vaccination-program.html
